# A child with multiple congenital anomalies due to partial trisomy 7q22.1 → qter resulting from a maternally inherited balanced translocation: a case report and review of literature

**DOI:** 10.1186/s12920-018-0366-6

**Published:** 2018-05-08

**Authors:** C. S. Paththinige, N. D. Sirisena, U. G. I. U. Kariyawasam, R. C. Ediriweera, P. Kruszka, M. Muenke, V. H. W. Dissanayake

**Affiliations:** 1 Human Genetics Unit, Faculty of Medicine, University of Colombo, Kynsey Road, Colombo, 00800 Sri Lanka; 2 Faculty of Medicine and Allied Sciences, Rajarata University of Sri Lanka, Saliyapura, Anuradhapura, 50008 Sri Lanka; 3 Lady Ridgeway Hospital for Children, Colombo, 00800 Sri Lanka; 4 Medical Genetics Branch, National Human Genome Research Institute, The National Institutes of Health, Bethesda, MD USA

**Keywords:** Congenital malformations, Translocation (7;14), Trisomy 7q, Fluorescence in-situ hybridization, SNP array

## Abstract

**Background:**

Parental balanced reciprocal translocations can result in partial aneuploidies in the offspring due to unbalanced meiotic segregation during gametogenesis. Herein, we report the phenotypic and molecular cytogenetic characterization of a 2 years and 4 months old female child with partial trisomy 7q22 → qter. This is the first such reported case resulting from a parental balanced translocation involving the long arms of chromosomes 7 and 14. The phenotype of the proband was compared with that of previously reported cases of trisomy 7q21 → qter or 7q22 → qter resulting from parental balanced translocations.

**Case presentation:**

The proband was born pre-term to a 34-year-old mother with a history of two first trimester miscarriages and an early infant death. She was referred at the age of 8 months for genetic evaluation due to prenatal and postnatal growth retardation, developmental delay and multiple congenital anomalies. On clinical evaluation, she had craniofacial dysmorphic features such as scaphocephaly, large anterior fontanelle with open posterior fontanelle, prominent occiput, triangular face, high forehead, hypertelorism, down slanting eyes, flat nasal bridge, small nose, low set ears, micro-retrognathia, high arched palate and short neck. Cranial computerized tomography scan showed lateral ventriculomegaly with features of early cerebral atrophy. Conventional cytogenetic analysis showed the karyotype 46,XX,der(14)t(7;14)(q22;q32)mat in the proband due to an unbalanced segregation of a maternal balanced translocation t(7;14)(q22;q32). Fluorescence in-situ hybridization analysis confirmed the partial trisomy 7q22 → qter in the proband with a minimal loss of genetic material on chromosome 14. Single nucleotide polymorphism array further confirmed the duplication on chromosome 7q22.1 → qter and a small terminal deletion on chromosome 14q32.3 → qter.

**Conclusion:**

We report the longest-surviving child with trisomy 7q22 → qter due to a parental balanced translocation between chromosomes 7 and 14. Clinical features observed in the proband were consistent with the consensus phenotype of partial trisomy 7q22 → qter reported in the scientific literature. Early diagnosis of these patients using molecular cytogenetic techniques is important for establishing the precise diagnosis and for making decisions pertaining to the prognostication and management of affected individuals.

**Electronic supplementary material:**

The online version of this article (10.1186/s12920-018-0366-6) contains supplementary material, which is available to authorized users.

## Background

Reciprocal translocations are structural chromosomal rearrangements, in which breakage of two chromosomes and exchange of the distal chromosomal segments occur, leading to the formation of two derivative chromosomes without any loss or gain of genetic material. These balanced translocations typically produce no significant phenotypic effects, unless one or both chromosomal breakpoints involve an important functional gene. However, during meiotic division they can undergo adjacent-1 or adjacent-2, 2:2 segregation, leading to the formation of gametes which are partially disomic for one chromosomal segment and partially nullisomic for the other. This results in a combination of partial trisomy and partial monosomy in the zygote.

Several cases of partial trisomy of long arm of chromosome 7 (7q) resulting from parental balanced translocations between chromosome 7 and another chromosome have been reported. The phenotype in such cases was attributed to the duplicated 7q chromosomal segment and the corresponding deleted segment of the other chromosome. However, the phenotypic effects of a large 7q duplication were considered to be of more importance than the smaller terminal deletion of the other chromosome [1]. Phenotypic features reported in partial trisomy 7q are somewhat non-specific and often seen in other chromosomal rearrangements as well, thus making phenotype-genotype correlation in this condition rather difficult [2].

Herein, we report a 2 years and 4 months old female child with pre- and postnatal growth retardation, developmental delay, and multiple congenital anomalies, who was found to have the karyotype 46,XX,der(14)t(7;14)(q22;q32)mat, resulting from a maternal balanced translocation, t(7;14)(q22;q32). Molecular cytogenetic testing confirmed trisomy 7q22.1 → qter in the proband. To the best of our knowledge, the occurrence of partial trisomy 7q22 → qter due to a parental balanced translocation involving the long arms of chromosomes 7 and 14 has not previously been reported.

## Case presentation

**Fig. 1:**
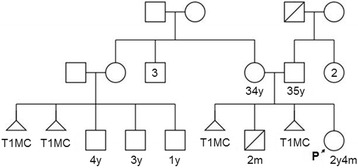
Pedigree of the proband. T1MC- first trimester miscarriage; Age- y: years, m: months

The proband was a female child born to a non-consanguineous couple; a 35-year-old father and a 34-year-old mother with a history of two first trimester miscarriages and an infant death at 2 months of age. The 3-generational pedigree of the proband is shown in Fig. 1. The first and third pregnancies of the proband’s mother ended in miscarriages at 7 and 8 weeks of gestation, respectively. The second pregnancy resulted in the birth of a full-term male child who developed respiratory arrest 1½ hours after birth followed by convulsions a day later. Ultrasonography of the brain showed cerebral oedema, and periventricular leukomalacia with a resolving intra-cranial hemorrhage. He later died at the age of 2 months. Neither an autopsy nor a genetic evaluation had been performed.

The CARE guidelines were followed in reporting the relevant information of the present case. The proband is the product of the fourth pregnancy. Routine prenatal scans had indicated intrauterine growth retardation. The baby was delivered by caesarean section at 35 weeks of gestation. Apgar scores were 9, 10 and 10 at 1, 5 and 10 min, respectively. The birth weight (1.5 kg) was below the 3rd centile expected for a baby born at 35 weeks of gestation and the head circumference (25.5 cm) and the crown to heel length (48 cm) were above the 50th centile. She developed respiratory distress within one hour postnatally and had to be resuscitated. She had a low hemoglobin level [11.3 g/dL (Normal > 14 g/dL)] with normal red cell indices, normal total and differential leukocyte counts and platelet counts. She was transfused on the fifth day after birth and post-transfusion hemoglobin level was 14.4 g/dl. Feeding difficulty due to poor sucking was noted at birth, but there was no record of muscular hypotonia.

At the age of 2 months, ophthalmological assessment showed pigmentary mottling of the retina, which later resolved. At the age of 5 months, bilateral lateral ventriculomegaly and periventricular thalamic striations were detected on ultrasound scan (USS) of the brain. USS of the abdomen did not show major anomalies except minimal free fluid observed in-between the loops of intestine. Echocardiography showed a structurally and functionally normal heart. Chest radiography was normal. Her thyroid profile and the full blood counts at follow-up visits were normal.

**Fig. 2:**
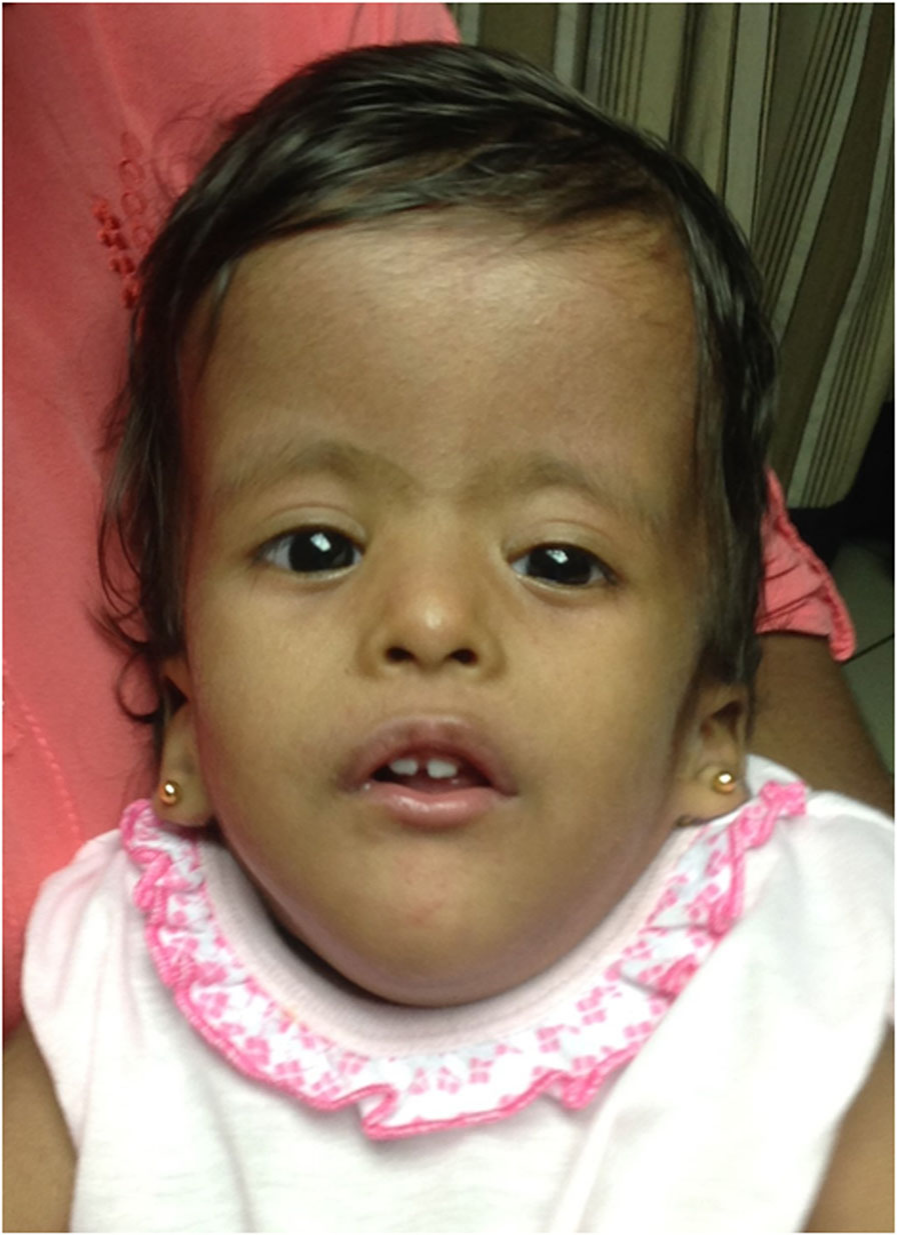
Facial photograph of the proband (frontal view) showing dysmorphic features such as triangular face, high forehead, hypertelorism, down slanting eyes, flat nasal bridge, small nose and low set ears

She was referred for genetic evaluation at the age of 8 months due to failure to thrive. Her weight and length at that time were 4.2 kg and 56 cm, respectively (both below 5th centile for the corrected age), and the head circumference was 41 cm (5th centile for the corrected age). Global developmental delay was noted. Several cranio-facial dysmorphic features were also noted such as scaphocephaly, large anterior fontanelle with open posterior fontanelle, prominent occiput, triangular face, high forehead, hypertelorism, down slanting eyes, flat nasal bridge, small nose, low set ears, micro-retrognathia, high arched palate, short neck, 5th finger clinodactyly and bilateral calcaneovarus (Fig. 2). No abnormalities were detected in the cardiovascular, respiratory and gastrointestinal systems. Hearing assessment was normal. She was managed with physiotherapy, language and speech therapy.

Computerized tomography scan of the brain at the age of 2 years showed prominent ventricular system with mild degree of periventricular hypo-densities which was suggestive of developing hydrocephalus. At the time of writing this report, the child is aged 2 years and 4 months. Her weight (6.1 kg) and height (75 cm) are below the 5th centile and the head circumference (46 cm) is at the 10th centile. She is able to sit with support, grasp objects with the palm and transfer them from hand to hand, respond to sounds, and make monosyllabic sounds. She is on regular follow-up at a child development clinic in a tertiary care hospital.

**Fig. 3:**
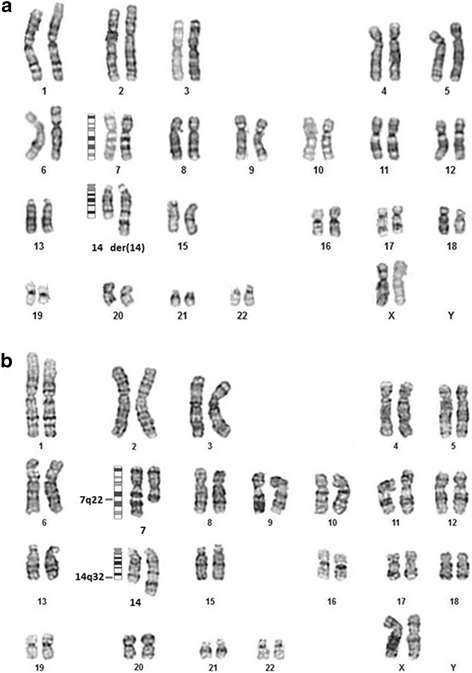
Karyograms (**a**) of the proband showing the derivative chromosome 14 and the normal chromosome 14 with the ideogram (**b**) of the proband’s mother showing the reciprocal translocation between chromosomes 7 and 14 with ideograms and the breakpoint on each chromosome

Chromosomal analysis of the proband’s peripheral venous blood using the GTL banding technique at 550 band resolution showed a derivative chromosome 14 in all 20 metaphase spreads analyzed (Fig. 3a). The mother’s chromosomal analysis using GTL banding technique at 500 band resolution showed a reciprocal translocation with the karyotype 46,XX,t(7;14)(q22;q32) (Fig. 3b). The karyotype of the proband was reported as 46,XX,der(14)t(7;14)(q22;q32)mat. Karyotype of the father showed normal chromosomal constitution.

**Fig. 4:**
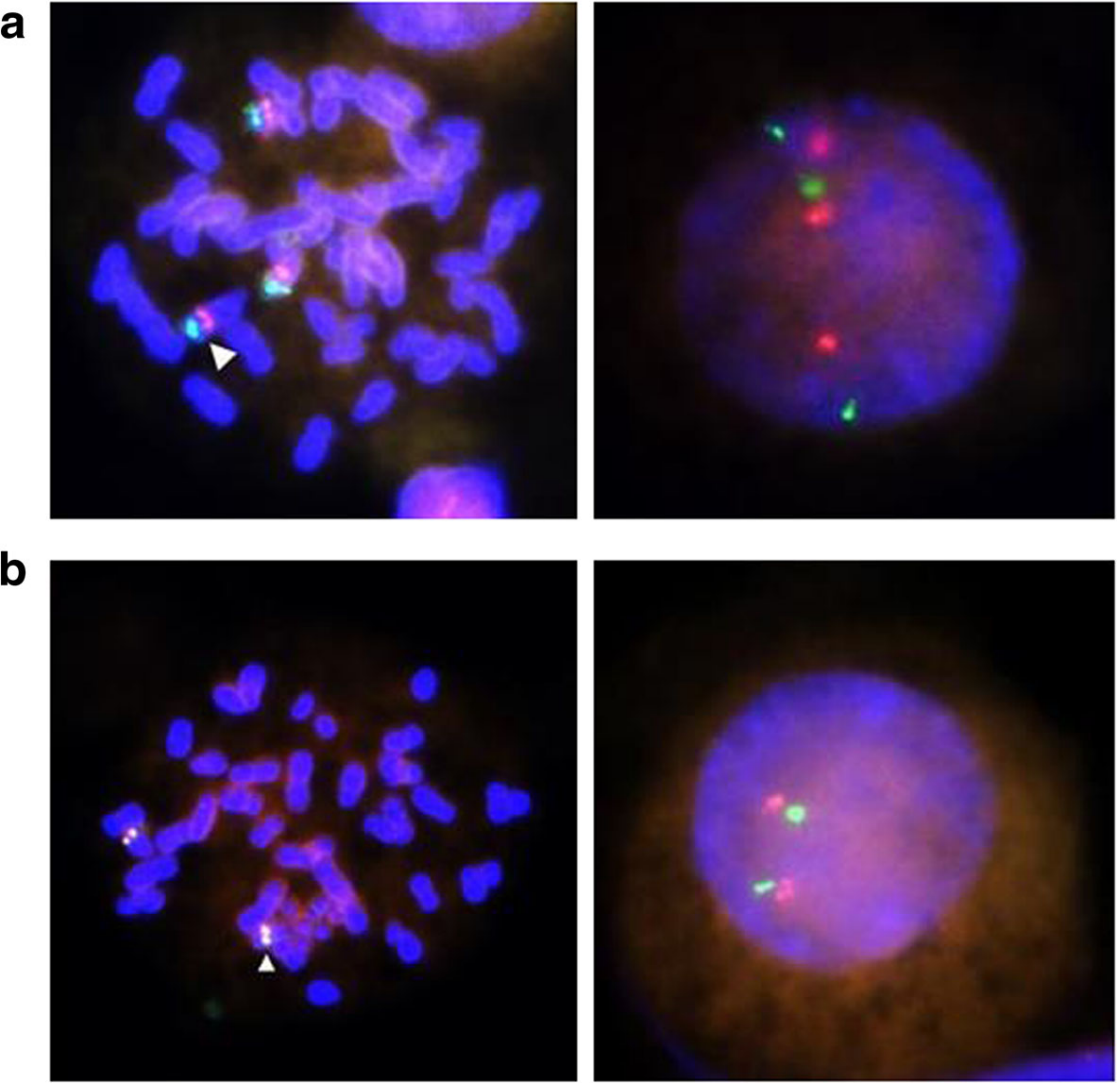
FISH analysis of the proband (**a**) Using the XL 7q22/7q36 locus specific probe (Metasystems, Altlussheim, Germany) showing hybridization signals on two normal chromosomes 7 and on the derivative chromosome 14 (indicated by the arrowhead) on metaphase chromosomes [Right] and on interphase chromosomes [Left]; Orange labelled probe hybridizes to 7q22 region including the *MLL5* gene. Green labelled probe hybridizes to 7q36 region that includes *EZH2* gene. **b** Using XL IGH plus probe (Metasystems, Altlussheim, Germany) hybridizing to constant region (orange) and variable distal region (green) of the *IGH* locus on chromosome 14 (14q32). Analysis shows normal hybridization signals on normal chromosome 14 and derivative chromosome 14 (indicated by the arrowhead) in metaphase chromosomes [Left] and interphase chromosomes [Right]

Fluorescence in-situ hybridization (FISH) of interphase and metaphase chromosomes was performed in the proband using locus specific probes for chromosomes 7 and 14. FISH analysis using XL 7q22/7q36 locus specific dual colour probe (7q22 including the *KMT2E* gene- spectrum orange and 7q36 including the *EZH2* gene- spectrum green) (Metasystems, Altlussheim, Germany) showed normal hybridization signals on both chromosomes 7. Additional hybridization signals for both 7q22 and 7q36 regions were seen on the derivative chromosome 14, indicating that the breakpoint in chromosome 7 is proximal to the *KMT2E* gene locus (chr7_105,014,190–105,114,085) in 7q22.3 region. This confirmed trisomy 7q22 → qter in the proband (Fig. 4a). FISH analysis of chromosome 14 was performed using XL IGH plus dual colour probe (Metasystems, Altlussheim, Germany) that hybridizes to the constant region of the IGH locus (spectrum orange) and variable distal region of IGH locus (spectrum green), both at 14q32.3. This showed normal hybridization signals (one green and one orange) on the normal chromosome 14 and the derivative chromosome 14 (Fig. 4b), indicating that the breakpoint in chromosome 14 is distal to the IGH locus (chr14_105,586,437–106,879,844).

**Fig. 5:**
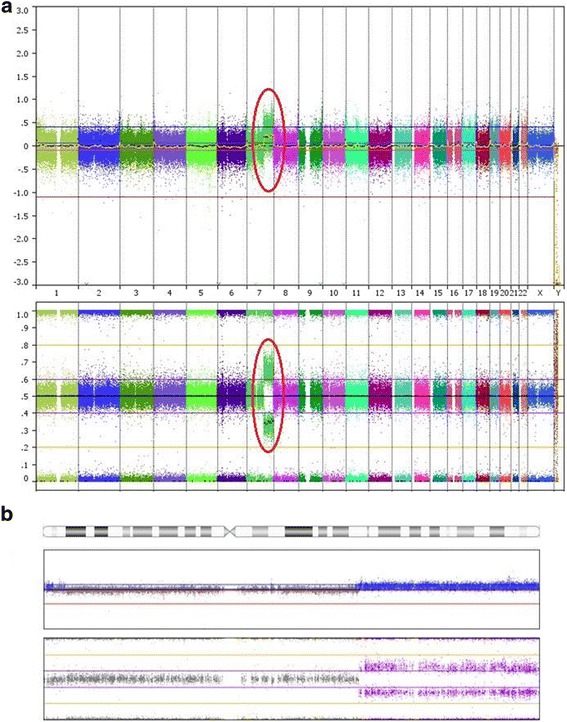
SNP array results of the proband (**a**) Full genome view of the proband, chromosome 7 duplication is circled. **b** Full view of chromosome 7. Top plots: Log R ratio representing the intensity data or the copy number, Bottom plots: B allele frequency

Further delineation of the chromosomal breakpoints and precise characterization of the duplicated and deleted chromosomal segments were done using single nucleotide polymorphism (SNP) array testing. The breakpoint on chromosome 7 was localized between chr7_101,105,923 and 101,150,073 (hg19). The first SNP showing an increase in intensity was located at chr7_101,139,424 and the last SNP of the array was located at chr7_159,119,486 confirming the duplication on chromosome 7q22.1 → qter, spanning 58 Mb (mega bases). The first deleted SNP on the chromosome 14 was chr14_106,948,749 and the deletion extended to the q terminus. This indicated a small (approximately 0.3 Mb in size) terminal deletion, 14q32.3 → qter on chromosome 14 (Fig. 5, Additional file 1).

## Discussion and conclusions

Due to the adjacent-1, 2:2 meiotic segregation of the maternal balanced chromosomal translocation, the proband was trisomic for a large segment of the long arm of chromosome 7 (7q22.1 → qter) with minimal loss of genetic material on chromosome 14. To the best of our knowledge, there are only three reports in the scientific literature that described a reciprocal translocation between chromosomes 7 and 14 in a parent resulting in partial aneuploidies in the offspring. One report described a baby with multiple congenital malformations, dysrhythmia and possible cardiac defect due to a familial reciprocal translocation involving the short arms of chromosome 7 (7p) and chromosome 14 (14p) [3]. In another case, partial monosomy 7q with concomitant partial trisomy14q was reported in a child with craniofacial and limb anomalies [4]. The third report described a baby with growth retardation, mental retardation and facial dysmorphism who was trisomic for 7q34 → qter region, due to a paternal reciprocal translocation t(7:14)(q34;q32) [5]. The present case is the first report of partial trisomy 7q22 → qter caused by a parental balanced translocation involving the long arms of chromosomes 7 and 14.

Partial trisomy 7q can occur either with or without a concomitant monosomy of another chromosome. Pure partial trisomy 7q results from the duplication of the whole or part of long arm of chromosome 7. Partial trisomy 7q with a monosomy of another chromosome was commonly caused by a parental reciprocal translocation involving the 7q segment. In cases of combined partial trisomy and partial monosomy with a proximal breakpoint in the long arm of chromosome 7 (7q), the breakpoint reported was either 7q21 or 7q22, which is similar to the present case. In 1982, Novales et al. described 3 distinct trisomy 7q syndromes; trisomy 7q21/22 → q31, trisomy 7q31 → qter and trisomy 7q32 → qter. Trisomy 7q31 → qter showed the most severe phenotypic manifestations and early postnatal death, while trisomy 7q21/22 → q31 had a milder phenotype with minor dysmorphic features and better survival [6]. A review of 5 cases with 7q21/22 → q31 duplication reported by Megarbane et al., showed that this category of patients had poorly defined phenotypic spectrum with non-specific cranio-facial dysmorphic features. Systemic malformations were uncommon in this group of patients [7]. In a recent compilation of 26 cases of interstitial duplications of chromosome 7 confined to 7q21 to 7q34 region, Rivera and Vasquez-Velasquez have also observed a relatively milder phenotypic expression with a longer lifespan in this group of patients with 7q duplication. Twenty four of the 26 cases reviewed by them had the proximal breakpoint at 7q21 or 7q22, further ratifying the common breakpoint in the long arm of chromosome 7 [8]. In 2001 Courtens et al. described a fourth group of trisomy 7q with the proximal breakpoint and extending to the 7q terminus (i.e. trisomy 7q21/22 → qter). The phenotypic features described in this group includes intrauterine growth retardation, hypotonia, facial dysmorphic features including micrognathia, cleft palate, genital and skeletal malformations, systemic malformations involving gastrointestinal and nervous systems and poor postnatal survival [1].

Phenotypic features and cytogenetic findings of our patient were compared with patients with partial trisomy 7q21 → qter or 7q22 → qter caused by a balanced parental translocation that were previously reported in the scientific literature [1, 9, 10] (Table 1). A common but non-specific group of clinical features were frequently observed among these patients. These features include intrauterine growth retardation, severe hypotonia, psychomotor retardation and dysmorphic features such as high forehead, hypertelorism, flat/wide nasal bridge, micro-retrognathia, low-set, malformed ears and short neck, conforming to the phenotype of 7q21/22 → qter syndrome described by Courtens et al. All these features except hypotonia were present in our patient. Her craniofacial profile with asymmetric skull, triangular facies, and micro-retrognathia is very similar to the ‘patient 2’ reported by Courtens et al. [1]. Respiratory distress at birth, reported in our patient is also a common feature in patients with partial trisomy 7q21/22 → qter, and has important clinical implications in the management of newborn babies with this structural chromosomal abnormality. Respiratory distress was also the cause of death in most affected babies who died in the early neonatal period or during infancy. Ventriculomegaly was a frequent finding in previously reported patients. Other cerebral and cerebellar malformations previously reported in these patients include cortical dysplasia and encephalomalacia. These cerebral and cerebellar malformations, although nonspecific, are prenatally detectable features of partial trisomy 7q21/22 → qter. A summary of the clinical features of the present case and the clinical features reported in cases with partial trisomy7q21/22 → qter due to parental balanced translocations is presented in Table 2.

**Table 1:** Comparison of present case with reported cases with trisomy 7q21 → qter/ 7q22 → qter due to parental balanced-translocations

	Forabosco et al., 1988 [9]			Courtens et al., 2001 [1]		Rodriguez et al., 2002 [10]	Present case
	Case 1	Case 2	Case 3	Case 1	Case 2		
Age	Newborn	Newborn	7 months	Fetus - 9 wks	Newborn	5 months	2 years and 4 months
Sex	F	M	F	F	F	F	F
Cytogenetic result							
- Trisomy	7q21-qter	7q21-qter	7q22-qter	7q21.2-qter	7q21.2-qter	7q21.2-qter, 15p	7q22.1-qter
- Monosomy	5p14-pter	13q33-qter	1qter	21p12-pter	4q35-qter	–	14q32.3-qter
- Parent	t(5;7)mat	t(7;13)pat	t(1;7)mat	t(7;21)mat	t(4;7)pat	t(7;15)mat	t(7;14)mat
Delivered at (weeks)	41	39	32	Termination	37 + 6 days	40	35 + 2 days
Birth weight	2200 g	2850 g	1950 g	(271 g)	1940 g	2.700 g	1500 g
Newborn respiratory distress	+	+	+	NA	+	+	+
Hypotonia	+	+	+			+	
Microcephaly	+	+					
Wide fontanelles	+		+	+		+	+
High forehead			+	+	+		+
Prominent occiput			+				+
Other skull anomalies					Asymmetry		Scaphocephaly
Triangular face					+		+
Hypertelorism	+	+	+	+	+		+
Flat/wide nasal bridge	+	+	+	+	+		+
Down slanting eyes	+	+	+		+		+
Other eye anomalies			L/Anoph-thalmia		Micro-ophthalmia		Retinal changes
Low-set ears	+	+	+	+	+		+
Malformed ears	+	+	+		+		
Small nose	+	+	+	+			+
Micro/retrognathia	+	+	+	+	+	+	+
Macroglossia		+	+		+		
Cleft palate		+		+	+	+	
High arched palate							+
Short neck	+	+	+	+	+		+
Low hairline					+ Posterior		+ Anterior
Clinodactyly					+		+
Skeletal deformities		Clubfoot				Scoliosis	Calcaneovarus
Cardiovascular defects				L/S SVC		PDA, HCM	
CNS malformations	+^a^		+^b^		+^c^	+^d^	+^e^
Abnormal EEG/ Seizures			+			+	
GI malformations				Malrotation	Complex^f^		
Mental retardation	NA	NA	+	NA	NA	+	+
Survival	38 h	23 h	7 months	Termination	2 days	11½ months	Alive

^a^dilated ventricles–mild hydrocephalus; ^b^diffuse hypodensity of cerebral white matter; ^c^focal cortical dysplasia of cerebellar vermis; ^d^dilatation of lateral ventricles, subependymal hemorrhage, multicystic encephalomalacia; ^e^bilateral lateral ventriculomegaly and periventricular thalamic striations; ^f^agenesis of appendix, hypoplasia of gallbladder, small and deformed pancreas, *NA* not applicable, *HCM* hypertrophic cardiomyopathy

**Table 2:** Summary of clinical features of the proband and the six patients reviewed in Table 1

Clinical features of the present case	No. of reported cases in the literature
Newborn respiratory distress	5
Wide fontanelle	4
Prominent occiput	1
Asymmetric skull	1
High forehead	3
Triangular face	1
Hypertelorism	5
Down slanting eyes	4
Flat/wide nasal bridge	5
Low set ears	5
Small/short nose	4
Micro-retrognathia	6
High arched palate	2
Short neck	5
Skeletal anomalies	2
CNS malformations	4
Psychomotor retardation	2^a^

^a^Only 2 babies survived beyond 2 days

We observed that there is considerable variability in the malformations involving other internal organs, but not the dysmorphic features among the patients with partial trisomy 7q21/22 → qter. Concomitant partial monosomy of the other chromosome could be an explanation for this variability, however the deleted chromosomal segment in our patient and in each of these cases reviewed were very small terminal segments and are unlikely to be the sole cause of this phenotypic variability. Reviews on trisomy 7q reported in the scientific literature have not shown a clear correlation between the phenotypic features (dysmorphic features and congenital malformations) and the genotype of the patients [2, 6]. This makes it difficult to predict the clinical outcome of affected patients either diagnosed prenatally or early in life based on their genotype.

In our patient, the only gene that is located within the presumed region of breakage of chromosome 7 (i.e. chr7_101,105,923–101,150,073) is the *SERPINE1* gene (chr7_101,127,087–101,139,266) encoding tissue plasminogen activator inhibitor protein. This gene lies in close proximity to the first SNP (chr7_101,139,424) that showed an increase in intensity in the SNP array of the proband. The *AP1S1* gene that encodes an adaptor protein involved in endocytosis and intracellular trafficking by regulating clarythrin coat assembly is located at chr7_101,154,405–101,161,276, just distal to the presumed region of breakage. Mutations of this gene are known to cause MEDNIK syndrome (MIM 609313) that is characterized by developmental delay, mental retardation, enteropathy, liver disorders and various neurological and cutaneous manifestations [11, 12].

The trisomic segment of chromosome 7 in our patient was approximately 58 Mb in size which constitutes over one third of the size of the chromosome 7. This region comprises of approximately 473 genes (Additional file 1), however the reports of possible candidate genes producing the phenotypic effects in patients with partial trisomy 7q are scarce in the scientific literature. One such recent report on *de-novo* 7q36.1q36.2 triplication suggested association of the increased dosage of *GALNT11* gene with multi-organ manifestations, due to alterations in the Notch signaling pathway that influence organogenesis and morphogenesis [13]. Another important gene in this region is the sonic hedgehog (*SHH*) gene (at 7q36) which codes for sonic hedgehog protein that is involved in the morphogenesis of the developing embryo. Aberrations in the sonic hedgehog signaling pathway due to alterations in *SHH* gene are implicated in congenital malformations involving the central nervous system and limbs [14–16]. The *SMO* gene encoding a G-protein coupled receptor that interacts with a receptor for hedgehog protein is also located in the duplicated region of chromosome 7 (at 7q32.1). Somatic mosaic mutations of this gene are implicated in the pathogenesis of Curry-Jones syndrome (MIM 601707) that is characterized by unicoronal craniosynostosis, cerebral malformations including ventriculomegaly, polysyndactyly, gastrointestinal and ocular anomalies [17]. In addition, several zinc-finger genes (*ZNF* genes) are located in the 7q22, 7q31 and 7q36 regions [18] within the duplicated chromosomal segment. It has been suggested that *ZNF* genes may contribute to the congenital malformations observed in complete or partial aneusomies [18, 19]. The *CNTNAP2* gene located in the 7q36 region is involved in cortical histogenesis and has been implicated in cortical dysplasia and neurodevelopmental disorders [20]. However, the effect of dosage imbalance of these genes due to partial trisomy 7q is yet to be described.

The deleted 14q32.3-qter chromosomal region of the proband contains only 3 genes; *LINC00221*, *MIR5195* and *MIR7641–2*. None of these genes have been associated with a significant phenotype. Moreover, there is no significant gene in the region of breakage of chromosome 14 in the proband. The closest gene with clinical relevance to the present case is the *BRF1* gene. Homozygous mutations in this gene are known to cause Cerebellofaciodental syndrome (MIM 616202) characterized by impaired cerebellar and cognitive development and dental and skeletal anomalies [21]. However, this gene is located 1.2 Mb proximal to the breakpoint on chromosome 14 in the proband. This suggests that the phenotypic features of the proband can be linked almost exclusively to the trisomy 7q22.1 → qter.

In summary**,** we described that partial trisomy 7q21 → qter or 7q22 → qter is characterized by a recognizable pattern of craniofacial dysmorphism and a range of congenital anomalies. This study corroborates the consensus phenotype associated with partial trisomy 7q22 → qter and further highlights its association with respiratory distress and cerebral malformations. Early diagnosis of these patients using cytogenetic testing is important to plan therapeutic measures with regards to the effective management of respiratory distress and neurological manifestations such as hypotonia, seizures and psychomotor retardation. Advanced molecular cytogenetic testing provides more conclusive information for the precise diagnosis and for making decisions pertaining to the prognostication and management of the affected individuals. More cases with similar chromosomal rearrangements need to be studied using molecular genetic techniques to identify a possible 7q triplication syndrome and determine its potential genetic aetiological mechanisms.

## Additional file

Additional file 1: SNP array datasheet of the proband and the parents. (XLSX 16 kb)

## Abbreviations

FISH: Fluorescence in-situ hybridization
SNP: Single nucleotide polymorphism
USS: Ultrasound scan

## References

[1] Courtens W, Vroman S, Vandenhove J, Wiedemann U, Schinzel A (2001). Pre-and perinatal findings in partial trisomy 7q resulting from balanced parental translocations t(7;21) and t(4;7). Prenatal Diag.

[2] Scelsa B, Bedeschi FM, Guerneri S, Lalatta F, Introvini P (2008). Partial trisomy of 7q: case report and literature review. J Child Neurol.

[3] Carnevale A, Frías S, Castillo VD (1978). Partial trisomy of the short arm of chromosome 7 due to a familial translocation rcp(7;14)(p11;p11). Clin Genet.

[4] Ponnala R, Dalal A (2011). Partial monosomy 7q. Indian Pediatr.

[5] Xiao B, Ji X, Jiang WT, Zhang JM, Hu Q, Tao J (2011). A case with partial trisomy 7 (q34→ qter) derived from a paternal reciprocal translocation t(7; 14)(q34; q32) [abstract]. Zhonghua yi xue yi chuan xue za zhi= Zhonghua yixue yichuanxue zazhi= Chinese J Med Genet.

[6] Novales MA, Fernandez-Novoa C, Hevia A, San Martin V, Galera H (1982). Partial trisomy for the long arm of chromosome 7. Case report and review. Hum Genet.

[7] Megarbane A, Gosset P, Souraty N, Lapierre JM, Turleau C, Vekemans M (2000). Chromosome 7q22-q31 duplication: report of a new case and review. Am J Med Genet.

[8] Rivera H, Vasquez-Velasquez AI (2013). De novo dup (7)(q21q22. 2) and cytogenetics of 7q21q34 duplications. Genet Couns.

[9] Forabosco A, Baroncini A, Dalpra L, Chessa L, Giannotti A, Maccagnani F, Dallapiccola B (1988). The phenotype of partial dup(7q) reconsidered: a report of five new cases. Clin Genet.

[10] Rodríguez L, López F, Paisán L, de la Red MD, Ruiz AM, Blanco M (2002). Pure partial trisomy 7q: two new patients and review. Am J Med Genet.

[11] Montpetit A, Côté S, Brustein E, Drouin CA, Lapointe L, Boudreau M (2008). Disruption of AP1S1, causing a novel neurocutaneous syndrome, perturbs development of the skin and spinal cord. PLoS Genet.

[12] Martinelli D, Travaglini L, Drouin CA, Ceballos-Picot I, Rizza T, Bertini E (2013). MEDNIK syndrome: a novel defect of copper metabolism treatable by zinc acetate therapy. Brain.

[13] El-Hattab AW, Allingham-Hawkins D, Al Dhaibani MA (2017). De novo chromosome 7q36. 1q36. 2 triplication in a child with developmental delay, growth failure, distinctive facial features, and multiple congenital anomalies: a case report. BMC Med Genet.

[14] Choudhry Z, Rikani AA, Choudhry AM, Tariq S, Zakaria F, Asghar MW (2014). Sonic hedgehog signaling pathway: a complex network. Ann Neurosci.

[15] Tickle C, Barker H (2013). The sonic hedgehog gradient in the developing limb. Wiley Interdisciplinary Reviews: Dev Biol.

[16] Lewis S (2013). Neural development: double agent sonic hedgehog. Nat Rev Neurosci.

[17] Twigg SR, Hufnagel RB, Miller KA, Zhou Y, McGowan SJ, Taylor J (2016). A recurrent mosaic mutation in SMO, encoding the hedgehog signal transducer smoothened, is the major cause of curry-Jones syndrome. Am J Hum Genet.

[18] Tommerup N, Vissing H (1995). Isolation and fine mapping of 16 novel human zinc finger-encoding cDNAs identify putative candidate genes for developmental and malignant disorders. Genomics.

[19] Tommerup N (1993). Mendelian cytogenetics. Chromosome rearrangements associated with mendelian disorders. J Med Genet.

[20] Strauss KA, Puffenberger EG, Huentelman MJ, Gottlieb S, Dobrin SE, Parod JM (2006). Recessive symptomatic focal epilepsy and mutant contactin-associated protein-like 2. N Engl J Med.

[21] Borck G, Hög F, Dentici ML, Tan PL, Sowada N, Medeira A (2015). BRF1 mutations alter RNA polymerase III–dependent transcription and cause neurodevelopmental anomalies. Genome Res.

